# Poly[[diaqua­(1,10-phenanthroline-κ^2^ *N*,*N*′)(μ_3_-4-sulfonato­benzene-1,2-dicarboxyl­ato-κ^4^ *O* ^1^:*O* ^2^ *,O* ^2′^:*O* ^4^)dysprosium(III)] dihydrate]

**DOI:** 10.1107/S1600536812003613

**Published:** 2012-02-04

**Authors:** Kou-Lin Zhang, Jian-Guo Lin, Seik Weng Ng

**Affiliations:** a College of Chemistry and Chemical Engineering, Yangzhou University, Yangzhou 225002, People’s Republic of China; b Department of Chemistry, University of Malaya, 50603 Kuala Lumpur, Malaysia; c Chemistry Department, King Abdulaziz University, PO Box 80203 Jeddah, Saudi Arabia

## Abstract

The 4-sulfophthalate trianion in the polymeric title complex, {[Dy(C_8_H_3_O_7_S)(C_12_H_8_N_2_)(H_2_O)_2_]·2H_2_O}_*n*_, bridges three water/phenanthroline-coordinated Dy^III^ atoms to form a three-dimensional network architecture. The metal atom is further chelated by a carboxyl­ate group and is covalently bonded to a monodentate carboxyl­ate group and to a monodentate sulfonate group in a distorted square-anti­prismatic geometry. The coordinating and the solvent water mol­ecules are hydrogen bonded to the network. In the crystal, one solvent water mol­ecule is disordered over two positions [major component = 59 (3)%].

## Related literature

For the isostructural Er^III^ complex, see: Zhang *et al.* (2012 ▶).
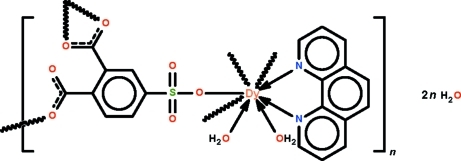


## Experimental

### 

#### Crystal data

[Dy(C_8_H_3_O_7_S)(C_12_H_8_N_2_)(H_2_O)_2_]·2H_2_O
*M* *_r_* = 657.93
Monoclinic, 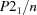

*a* = 14.3852 (7) Å
*b* = 9.6487 (5) Å
*c* = 17.4280 (9) Å
β = 105.770 (1)°
*V* = 2327.9 (2) Å^3^
*Z* = 4
Mo *K*α radiation
μ = 3.36 mm^−1^
*T* = 293 K
0.50 × 0.30 × 0.20 mm

#### Data collection

Bruker SMART APEX diffractometer
Absorption correction: multi-scan (*SADABS*; Sheldrick, 1996 ▶) *T*_min_ = 0.473, *T*_max_ = 1.000
6452 measured reflections
4022 independent reflections
3864 reflections with *I* > 2σ(*I*)
*R*_int_ = 0.023

#### Refinement

*R*[*F* ^2^ > 2σ(*F* ^2^)] = 0.037
*wR*(*F* ^2^) = 0.093
*S* = 1.20
4022 reflections
353 parameters
33 restraints
H atoms treated by a mixture of independent and constrained refinement
Δρ_max_ = 0.97 e Å^−3^
Δρ_min_ = −1.85 e Å^−3^

### 

Data collection: *APEX2* (Bruker, 2005 ▶); cell refinement: *SAINT* (Bruker, 2005 ▶); data reduction: *SAINT*; program(s) used to solve structure: *SHELXS97* (Sheldrick, 2008 ▶); program(s) used to refine structure: *SHELXL97* (Sheldrick, 2008 ▶); molecular graphics: *X-SEED* (Barbour, 2001 ▶); software used to prepare material for publication: *publCIF* (Westrip, 2010 ▶).

## Supplementary Material

Crystal structure: contains datablock(s) global, I. DOI: 10.1107/S1600536812003613/xu5457sup1.cif

Structure factors: contains datablock(s) I. DOI: 10.1107/S1600536812003613/xu5457Isup2.hkl

Additional supplementary materials:  crystallographic information; 3D view; checkCIF report

## Figures and Tables

**Table 1:** Hydrogen-bond geometry (Å, °)

*D*—H⋯*A*	*D*—H	H⋯*A*	*D*⋯*A*	*D*—H⋯*A*
O1*w*—H11⋯O5^i^	0.84 (1)	1.98 (1)	2.817 (6)	175 (7)
O1*w*—H12⋯O7^ii^	0.84 (1)	1.94 (2)	2.760 (6)	166 (6)
O2*w*—H21⋯O2	0.84 (1)	1.96 (3)	2.744 (7)	156 (7)
O2*w*—H22⋯O3*w*	0.84 (1)	1.83 (2)	2.66 (1)	169 (7)
O3*W*—H31⋯O7^iii^	0.84 (1)	2.05 (1)	2.81 (1)	151 (2)
O3*w*′—H33⋯O7^iii^	0.84 (1)	2.05 (1)	2.74 (2)	139 (2)
O4*w*—H41⋯O2^iv^	0.84 (1)	2.11 (5)	2.897 (8)	155 (11)
O4*w*—H41⋯O2^iv^	0.84 (1)	2.11 (5)	2.897 (8)	155 (11)
O4*w*—H42⋯O3^v^	0.84 (1)	2.04 (6)	2.787 (9)	148 (11)

Symmetry codes: (i) 
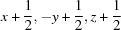
; (ii) 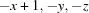; (iii) 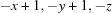; (iv) 
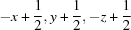
; (v) 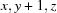.

**Fig. 1.**
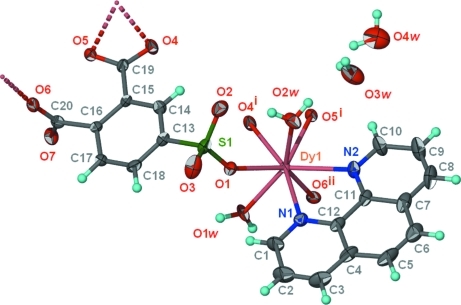
Thermal ellipsoid plot (Barbour, 2001) of the formula unit of polymeric [Dy(H2O)2(C12H8N2)(C8H3O7S)]n.2nH2O at the 50% probability level; hydrogen atoms are drawn as spheres of arbitrary radius. The disorder is not shown.

## supplementary crystallographic information

### Comment

The deprotonated 4-sulfophthalic acid trianion forms a number of coordination polymers as its carboxyl and sulfo groups are capable of a variety of bonding modes. Among these, the 1,10-phenanthroline-coordinated europium derivative exists as a monoaqua coordination polymer adopting a chain motif. The Er^III^ analog is instead a diaqua coordination polymer adopting a three-dimensional network motif. The title Dy_III_ analog is isostructural with the Er^III^ derivative (Zhang *et al.*, 2012). The 4-sulfophthalate trianion bridges three water/phenanthroline-coordinated Dy^III^ atoms to form a three-dimensional network architecture (Scheme I, Fig. 1). The metal atom is chelated by a carboxyl group and is covalently bonded to a unidentate carboxyl as well as to a unidentate sulfo group in a square antiprismatic geometry. The lattice water molecules are hydrogen-bonded to the network. Other O–H···O hydrogen bonds are also present (Table 1).

### Experimental

4-Sulfophthalic acid (0.080 g), 1,10-phenanthroline (0.057 g), erbium trichloride hexahydrate (0.113 g) and water (10 ml) were placed in a 25 -ml Teflon-lined stainless-steel Parr bomb. The vessel was heated at 443 K for 3 days. Red crystals were obtained when the vessel was cooled to room temperature slowly in about 30% yield.

### Refinement

Carbon-bound H-atoms were placed in calculated positions (C—H 0.93 Å) and were included in the refinement in the riding model approximation, with *U*(H) set to 1.2*U*(C).

The water H-atoms were located in a difference Fourier map, and were refined with distance restraints of O–H 0.84±0.01 and H···H 1.37±0.01 Å; their temperature factors were tied by a factor of 1.5 times.

The O3w water molecule is disordered over tw sites in a 0.59 (3): 0.4135 ratio. The disorder components share a common H atom, which forms a hydrogen bond to an acceptor atom.

The anisotropic temperature factors of the lattice water O atoms were tightly restrained to be nearly isotropic.

The final difference Fourier map had a peak at 0.90 Å from Dy1 and a hole at 0.86 Å from this heavy atom.

### Crystal data

**Table:**

[Dy(C_8_H_3_O_7_S)(C_12_H_8_N_2_)(H_2_O)_2_]·2H_2_O	*F*(000) = 1292
*M**_r_* = 657.93	*D*_x_ = 1.877 Mg m^−^^3^
Monoclinic, *P*2_1_/*n*	Mo *K*α radiation, λ = 0.71073 Å
Hall symbol: -P 2yn	Cell parameters from 5412 reflections
*a* = 14.3852 (7) Å	θ = 2.2–25.1°
*b* = 9.6487 (5) Å	µ = 3.36 mm^−^^1^
*c* = 17.4280 (9) Å	*T* = 293 K
β = 105.770 (1)°	Block, red
*V* = 2327.9 (2) Å^3^	0.50 × 0.30 × 0.20 mm
*Z* = 4	

### Data collection

**Table:**

Bruker SMART APEX diffractometer	4022 independent reflections
Radiation source: fine-focus sealed tube	3864 reflections with *I* > 2σ(*I*)
Graphite monochromator	*R*_int_ = 0.023
ω scans	θ_max_ = 25.1°, θ_min_ = 2.2°
Absorption correction: multi-scan (*SADABS*; Sheldrick, 1996)	*h* = −17→16
*T*_min_ = 0.473, *T*_max_ = 1.000	*k* = −11→9
6452 measured reflections	*l* = −20→19

### Refinement

**Table:**

Refinement on *F*^2^	Primary atom site location: structure-invariant direct methods
Least-squares matrix: full	Secondary atom site location: difference Fourier map
*R*[*F*^2^ > 2σ(*F*^2^)] = 0.037	Hydrogen site location: inferred from neighbouring sites
*wR*(*F*^2^) = 0.093	H atoms treated by a mixture of independent and constrained refinement
*S* = 1.20	*w* = 1/[σ^2^(*F*_o_^2^) + (0.0378*P*)^2^ + 10.2868*P*] where *P* = (*F*_o_^2^ + 2*F*_c_^2^)/3
4022 reflections	(Δ/σ)_max_ = 0.001
353 parameters	Δρ_max_ = 0.97 e Å^−^^3^
33 restraints	Δρ_min_ = −1.85 e Å^−^^3^

### Special details

**Table:**

Geometry. All e.s.d.'s (except the e.s.d. in the dihedral angle between two l.s. planes) are estimated using the full covariance matrix. The cell e.s.d.'s are taken into account individually in the estimation of e.s.d.'s in distances, angles and torsion angles; correlations between e.s.d.'s in cell parameters are only used when they are defined by crystal symmetry. An approximate (isotropic) treatment of cell e.s.d.'s is used for estimating e.s.d.'s involving l.s. planes.

### Fractional atomic coordinates and isotropic or equivalent isotropic displacement parameters (Å^2^)

**Table:**

	*x*	*y*	*z*	*U*_iso_*/*U*_eq_	Occ. (<1)
Dy1	0.604921 (17)	0.47289 (2)	0.259769 (14)	0.02131 (11)	
S1	0.36749 (10)	0.32357 (16)	0.16213 (8)	0.0313 (3)	
O1	0.4682 (3)	0.3307 (4)	0.2104 (2)	0.0319 (9)	
O2	0.3258 (3)	0.4612 (5)	0.1434 (3)	0.0473 (12)	
O3	0.3103 (4)	0.2348 (6)	0.1978 (3)	0.0562 (14)	
O4	0.4063 (4)	0.4842 (4)	−0.1216 (3)	0.0452 (12)	
O5	0.3528 (3)	0.3141 (4)	−0.2043 (2)	0.0292 (8)	
O6	0.2670 (3)	0.0319 (4)	−0.1959 (2)	0.0303 (9)	
O7	0.4245 (3)	−0.0030 (5)	−0.1685 (3)	0.0400 (10)	
O1w	0.6545 (3)	0.2479 (4)	0.2389 (3)	0.0390 (10)	
H11	0.7126 (15)	0.224 (6)	0.256 (4)	0.059*	
H12	0.622 (4)	0.180 (4)	0.217 (4)	0.059*	
O2w	0.4682 (3)	0.6129 (5)	0.2456 (3)	0.0432 (11)	
H21	0.422 (4)	0.589 (7)	0.207 (3)	0.065*	
H22	0.465 (5)	0.6988 (19)	0.253 (4)	0.065*	
O3w	0.4836 (11)	0.8837 (11)	0.2752 (11)	0.069 (4)	0.59 (3)
H31	0.492 (4)	0.911 (11)	0.2318 (17)	0.103*	0.59 (3)
H32	0.523 (10)	0.923 (16)	0.313 (2)	0.103*	0.59 (3)
O3w'	0.4379 (15)	0.8711 (16)	0.2234 (15)	0.062 (6)	0.41 (3)
H33	0.492 (4)	0.911 (11)	0.2318 (17)	0.093*	0.41 (3)
H34	0.417 (10)	0.85 (3)	0.174 (3)	0.093*	0.41 (3)
O4w	0.3507 (5)	1.0315 (7)	0.3158 (4)	0.0733 (18)	
H41	0.304 (5)	0.986 (11)	0.323 (6)	0.110*	
H42	0.335 (7)	1.064 (12)	0.269 (3)	0.110*	
N1	0.5893 (4)	0.3636 (5)	0.3864 (3)	0.0321 (11)	
N2	0.6436 (4)	0.6324 (5)	0.3796 (3)	0.0334 (11)	
C1	0.5629 (5)	0.2329 (7)	0.3899 (4)	0.0439 (16)	
H1	0.5472	0.1816	0.3430	0.053*	
C2	0.5574 (6)	0.1671 (8)	0.4602 (5)	0.057 (2)	
H2	0.5386	0.0749	0.4598	0.068*	
C3	0.5804 (5)	0.2422 (9)	0.5294 (4)	0.0536 (19)	
H3	0.5777	0.2006	0.5769	0.064*	
C4	0.6079 (4)	0.3812 (8)	0.5291 (4)	0.0409 (16)	
C5	0.6328 (5)	0.4680 (10)	0.5983 (4)	0.054 (2)	
H5	0.6293	0.4317	0.6469	0.065*	
C6	0.6607 (5)	0.5984 (10)	0.5956 (4)	0.055 (2)	
H6	0.6764	0.6508	0.6421	0.066*	
C7	0.6673 (5)	0.6603 (8)	0.5226 (4)	0.0426 (16)	
C8	0.6981 (6)	0.7960 (9)	0.5163 (5)	0.060 (2)	
H8	0.7165	0.8514	0.5616	0.072*	
C9	0.7016 (6)	0.8474 (9)	0.4453 (5)	0.064 (2)	
H9	0.7225	0.9377	0.4414	0.076*	
C10	0.6732 (5)	0.7635 (7)	0.3775 (4)	0.0464 (17)	
H10	0.6751	0.8004	0.3286	0.056*	
C11	0.6409 (4)	0.5806 (7)	0.4521 (3)	0.0324 (13)	
C12	0.6114 (4)	0.4385 (7)	0.4552 (3)	0.0306 (12)	
C13	0.3692 (4)	0.2467 (6)	0.0699 (3)	0.0288 (12)	
C14	0.3764 (4)	0.3288 (6)	0.0069 (3)	0.0292 (12)	
H14	0.3835	0.4242	0.0136	0.035*	
C15	0.3730 (4)	0.2696 (6)	−0.0663 (3)	0.0268 (11)	
C16	0.3638 (4)	0.1247 (6)	−0.0757 (3)	0.0245 (11)	
C17	0.3649 (4)	0.0425 (6)	−0.0092 (3)	0.0305 (12)	
H17	0.3652	−0.0535	−0.0134	0.037*	
C18	0.3655 (4)	0.1044 (7)	0.0627 (3)	0.0340 (13)	
H18	0.3635	0.0498	0.1061	0.041*	
C19	0.3777 (4)	0.3615 (6)	−0.1346 (3)	0.0295 (12)	
C20	0.3526 (4)	0.0487 (6)	−0.1538 (3)	0.0274 (12)	

### Atomic displacement parameters (Å^2^)

**Table:**

	*U*^11^	*U*^22^	*U*^33^	*U*^12^	*U*^13^	*U*^23^
Dy1	0.02671 (16)	0.01852 (16)	0.01882 (15)	−0.00121 (9)	0.00642 (10)	−0.00062 (9)
S1	0.0288 (7)	0.0446 (9)	0.0204 (7)	−0.0047 (6)	0.0065 (5)	−0.0014 (6)
O1	0.029 (2)	0.032 (2)	0.028 (2)	−0.0017 (16)	−0.0031 (16)	−0.0001 (17)
O2	0.045 (3)	0.057 (3)	0.037 (3)	0.019 (2)	0.005 (2)	−0.004 (2)
O3	0.055 (3)	0.083 (4)	0.036 (3)	−0.033 (3)	0.023 (2)	−0.012 (3)
O4	0.084 (4)	0.027 (2)	0.027 (2)	−0.014 (2)	0.019 (2)	−0.0056 (18)
O5	0.042 (2)	0.024 (2)	0.0197 (19)	−0.0047 (16)	0.0042 (16)	−0.0012 (15)
O6	0.024 (2)	0.028 (2)	0.035 (2)	−0.0025 (15)	0.0009 (17)	−0.0058 (16)
O7	0.035 (2)	0.037 (2)	0.049 (3)	0.0041 (19)	0.012 (2)	−0.009 (2)
O1w	0.033 (2)	0.024 (2)	0.058 (3)	0.0032 (17)	0.010 (2)	−0.011 (2)
O2w	0.042 (3)	0.029 (2)	0.057 (3)	0.0053 (19)	0.012 (2)	0.000 (2)
O3w	0.082 (8)	0.048 (5)	0.084 (9)	0.005 (5)	0.035 (7)	0.004 (5)
O3w'	0.066 (9)	0.043 (7)	0.081 (10)	0.012 (6)	0.027 (7)	0.011 (6)
O4w	0.078 (4)	0.077 (4)	0.077 (4)	−0.001 (3)	0.042 (3)	0.010 (3)
N1	0.040 (3)	0.032 (3)	0.025 (2)	−0.003 (2)	0.009 (2)	−0.001 (2)
N2	0.041 (3)	0.032 (3)	0.029 (3)	−0.005 (2)	0.011 (2)	−0.009 (2)
C1	0.060 (4)	0.037 (4)	0.037 (4)	−0.006 (3)	0.018 (3)	0.005 (3)
C2	0.069 (5)	0.048 (4)	0.059 (5)	−0.006 (4)	0.027 (4)	0.021 (4)
C3	0.055 (4)	0.073 (5)	0.038 (4)	0.007 (4)	0.022 (3)	0.024 (4)
C4	0.031 (3)	0.066 (5)	0.026 (3)	0.008 (3)	0.009 (2)	0.008 (3)
C5	0.047 (4)	0.095 (7)	0.021 (3)	0.015 (4)	0.010 (3)	0.006 (3)
C6	0.046 (4)	0.083 (6)	0.031 (4)	0.008 (4)	0.004 (3)	−0.021 (4)
C7	0.034 (3)	0.062 (5)	0.032 (3)	0.006 (3)	0.009 (3)	−0.012 (3)
C8	0.071 (5)	0.066 (5)	0.045 (4)	−0.015 (4)	0.019 (4)	−0.032 (4)
C9	0.084 (6)	0.049 (5)	0.065 (5)	−0.017 (4)	0.032 (5)	−0.033 (4)
C10	0.067 (5)	0.036 (4)	0.042 (4)	−0.016 (3)	0.024 (3)	−0.012 (3)
C11	0.030 (3)	0.044 (4)	0.023 (3)	−0.002 (3)	0.007 (2)	−0.008 (3)
C12	0.023 (3)	0.045 (3)	0.023 (3)	0.006 (2)	0.006 (2)	0.001 (2)
C13	0.027 (3)	0.038 (3)	0.021 (3)	−0.003 (2)	0.005 (2)	−0.002 (2)
C14	0.038 (3)	0.026 (3)	0.024 (3)	−0.003 (2)	0.008 (2)	−0.001 (2)
C15	0.032 (3)	0.024 (3)	0.023 (3)	−0.003 (2)	0.006 (2)	0.003 (2)
C16	0.021 (3)	0.028 (3)	0.021 (3)	0.000 (2)	0.000 (2)	0.002 (2)
C17	0.031 (3)	0.026 (3)	0.030 (3)	0.002 (2)	0.002 (2)	0.007 (2)
C18	0.033 (3)	0.042 (4)	0.027 (3)	−0.004 (3)	0.007 (2)	0.010 (3)
C19	0.038 (3)	0.025 (3)	0.027 (3)	0.001 (2)	0.011 (2)	0.001 (2)
C20	0.030 (3)	0.021 (3)	0.031 (3)	−0.001 (2)	0.007 (2)	−0.001 (2)

### Geometric parameters (Å, º)

**Table:**

Dy1—O6^i^	2.249 (4)	N2—C11	1.368 (8)
Dy1—O2w	2.343 (4)	C1—C2	1.401 (9)
Dy1—O1w	2.344 (4)	C1—H1	0.9300
Dy1—O1	2.360 (4)	C2—C3	1.368 (12)
Dy1—O4^ii^	2.406 (4)	C2—H2	0.9300
Dy1—O5^ii^	2.418 (4)	C3—C4	1.399 (11)
Dy1—N1	2.511 (5)	C3—H3	0.9300
Dy1—N2	2.532 (5)	C4—C12	1.415 (8)
S1—O3	1.440 (5)	C4—C5	1.432 (10)
S1—O2	1.458 (5)	C5—C6	1.325 (12)
S1—O1	1.466 (4)	C5—H5	0.9300
S1—C13	1.776 (6)	C6—C7	1.432 (11)
O4—C19	1.253 (7)	C6—H6	0.9300
O4—Dy1^ii^	2.406 (4)	C7—C8	1.396 (11)
O5—C19	1.256 (7)	C7—C11	1.411 (8)
O5—Dy1^ii^	2.418 (4)	C8—C9	1.346 (12)
O6—C20	1.260 (7)	C8—H8	0.9300
O6—Dy1^iii^	2.249 (4)	C9—C10	1.400 (10)
O7—C20	1.236 (7)	C9—H9	0.9300
O1w—H11	0.840 (10)	C10—H10	0.9300
O1w—H12	0.839 (10)	C11—C12	1.441 (9)
O2w—H21	0.840 (10)	C13—C18	1.378 (9)
O2w—H22	0.840 (10)	C13—C14	1.380 (8)
O3w—H31	0.838 (10)	C14—C15	1.386 (8)
O3w—H32	0.838 (11)	C14—H14	0.9300
O3w—H33	0.838 (10)	C15—C16	1.410 (8)
O3w'—H31	0.842 (10)	C15—C19	1.502 (7)
O3w'—H33	0.842 (10)	C16—C17	1.399 (8)
O3w'—H34	0.840 (10)	C16—C20	1.516 (8)
O4w—H41	0.842 (10)	C17—C18	1.386 (9)
O4w—H42	0.842 (10)	C17—H17	0.9300
N1—C1	1.323 (8)	C18—H18	0.9300
N1—C12	1.361 (7)	C19—Dy1^ii^	2.769 (6)
N2—C10	1.338 (8)		
			
O6^i^—Dy1—O2w	144.11 (16)	C3—C2—H2	120.9
O6^i^—Dy1—O1w	72.76 (14)	C1—C2—H2	120.9
O2w—Dy1—O1w	143.10 (16)	C2—C3—C4	120.3 (6)
O6^i^—Dy1—O1	142.83 (14)	C2—C3—H3	119.8
O2w—Dy1—O1	72.81 (16)	C4—C3—H3	119.8
O1w—Dy1—O1	70.34 (14)	C3—C4—C12	117.3 (6)
O6^i^—Dy1—O4^ii^	97.40 (17)	C3—C4—C5	124.3 (6)
O2w—Dy1—O4^ii^	88.24 (18)	C12—C4—C5	118.4 (7)
O1w—Dy1—O4^ii^	86.81 (16)	C6—C5—C4	122.3 (7)
O1—Dy1—O4^ii^	84.97 (15)	C6—C5—H5	118.9
O6^i^—Dy1—O5^ii^	78.73 (14)	C4—C5—H5	118.9
O2w—Dy1—O5^ii^	76.29 (15)	C5—C6—C7	121.5 (7)
O1w—Dy1—O5^ii^	127.22 (15)	C5—C6—H6	119.3
O1—Dy1—O5^ii^	128.57 (13)	C7—C6—H6	119.3
O4^ii^—Dy1—O5^ii^	53.80 (13)	C8—C7—C11	117.3 (6)
O6^i^—Dy1—N1	91.24 (15)	C8—C7—C6	124.1 (7)
O2w—Dy1—N1	93.53 (17)	C11—C7—C6	118.6 (7)
O1w—Dy1—N1	81.44 (17)	C9—C8—C7	120.6 (7)
O1—Dy1—N1	79.13 (15)	C9—C8—H8	119.7
O4^ii^—Dy1—N1	162.68 (15)	C7—C8—H8	119.7
O5^ii^—Dy1—N1	143.20 (14)	C8—C9—C10	119.2 (8)
O6^i^—Dy1—N2	75.75 (16)	C8—C9—H9	120.4
O2w—Dy1—N2	74.13 (17)	C10—C9—H9	120.4
O1w—Dy1—N2	133.09 (17)	N2—C10—C9	123.0 (7)
O1—Dy1—N2	128.97 (15)	N2—C10—H10	118.5
O4^ii^—Dy1—N2	131.39 (15)	C9—C10—H10	118.5
O5^ii^—Dy1—N2	77.89 (15)	N2—C11—C7	122.4 (6)
N1—Dy1—N2	65.32 (16)	N2—C11—C12	117.7 (5)
O3—S1—O2	113.0 (3)	C7—C11—C12	119.9 (6)
O3—S1—O1	111.8 (3)	N1—C12—C4	122.4 (6)
O2—S1—O1	111.6 (3)	N1—C12—C11	118.2 (5)
O3—S1—C13	107.0 (3)	C4—C12—C11	119.3 (6)
O2—S1—C13	106.5 (3)	C18—C13—C14	120.5 (5)
O1—S1—C13	106.5 (3)	C18—C13—S1	119.3 (4)
S1—O1—Dy1	146.3 (3)	C14—C13—S1	120.2 (5)
C19—O4—Dy1^ii^	92.9 (3)	C13—C14—C15	120.3 (5)
C19—O5—Dy1^ii^	92.3 (3)	C13—C14—H14	119.9
C20—O6—Dy1^iii^	163.8 (4)	C15—C14—H14	119.9
Dy1—O1w—H11	121 (4)	C14—C15—C16	119.6 (5)
Dy1—O1w—H12	130 (4)	C14—C15—C19	119.3 (5)
H11—O1w—H12	109.6 (18)	C16—C15—C19	121.1 (5)
Dy1—O2w—H21	113 (5)	C17—C16—C15	119.1 (5)
Dy1—O2w—H22	129 (5)	C17—C16—C20	116.3 (5)
H21—O2w—H22	109 (2)	C15—C16—C20	124.6 (5)
H31—O3w—H32	110 (2)	C18—C17—C16	120.0 (5)
H32—O3w—H33	110 (2)	C18—C17—H17	120.0
H31—O3w'—H34	109 (2)	C16—C17—H17	120.0
H33—O3w'—H34	109 (2)	C13—C18—C17	120.2 (5)
H41—O4w—H42	109 (2)	C13—C18—H18	119.9
C1—N1—C12	117.8 (5)	C17—C18—H18	119.9
C1—N1—Dy1	122.4 (4)	O4—C19—O5	120.9 (5)
C12—N1—Dy1	119.7 (4)	O4—C19—C15	119.8 (5)
C10—N2—C11	117.4 (5)	O5—C19—C15	119.3 (5)
C10—N2—Dy1	123.5 (4)	O4—C19—Dy1^ii^	60.2 (3)
C11—N2—Dy1	118.9 (4)	O5—C19—Dy1^ii^	60.8 (3)
N1—C1—C2	123.9 (7)	C15—C19—Dy1^ii^	177.4 (4)
N1—C1—H1	118.1	O7—C20—O6	124.6 (5)
C2—C1—H1	118.1	O7—C20—C16	119.4 (5)
C3—C2—C1	118.3 (7)	O6—C20—C16	115.7 (5)
			
O3—S1—O1—Dy1	−140.2 (4)	C8—C9—C10—N2	−0.8 (13)
O2—S1—O1—Dy1	−12.6 (6)	C10—N2—C11—C7	0.4 (9)
C13—S1—O1—Dy1	103.2 (5)	Dy1—N2—C11—C7	176.0 (4)
O6^i^—Dy1—O1—S1	−155.1 (4)	C10—N2—C11—C12	−178.5 (6)
O2w—Dy1—O1—S1	30.2 (4)	Dy1—N2—C11—C12	−2.8 (7)
O1w—Dy1—O1—S1	−147.9 (5)	C8—C7—C11—N2	−0.8 (10)
O4^ii^—Dy1—O1—S1	−59.5 (5)	C6—C7—C11—N2	179.0 (6)
O5^ii^—Dy1—O1—S1	−25.5 (5)	C8—C7—C11—C12	178.0 (6)
N1—Dy1—O1—S1	127.4 (5)	C6—C7—C11—C12	−2.2 (9)
N2—Dy1—O1—S1	82.0 (5)	C1—N1—C12—C4	0.5 (9)
O6^i^—Dy1—N1—C1	−106.3 (5)	Dy1—N1—C12—C4	−177.3 (4)
O2w—Dy1—N1—C1	109.3 (5)	C1—N1—C12—C11	179.5 (6)
O1w—Dy1—N1—C1	−33.9 (5)	Dy1—N1—C12—C11	1.7 (7)
O1—Dy1—N1—C1	37.6 (5)	C3—C4—C12—N1	0.0 (9)
O4^ii^—Dy1—N1—C1	13.9 (9)	C5—C4—C12—N1	179.8 (6)
O5^ii^—Dy1—N1—C1	−179.0 (4)	C3—C4—C12—C11	−179.0 (6)
N2—Dy1—N1—C1	−179.9 (6)	C5—C4—C12—C11	0.8 (8)
O6^i^—Dy1—N1—C12	71.5 (4)	N2—C11—C12—N1	0.8 (8)
O2w—Dy1—N1—C12	−72.9 (4)	C7—C11—C12—N1	−178.1 (5)
O1w—Dy1—N1—C12	143.9 (4)	N2—C11—C12—C4	179.8 (5)
O1—Dy1—N1—C12	−144.6 (4)	C7—C11—C12—C4	1.0 (8)
O4^ii^—Dy1—N1—C12	−168.3 (5)	O3—S1—C13—C18	−31.9 (6)
O5^ii^—Dy1—N1—C12	−1.2 (6)	O2—S1—C13—C18	−153.0 (5)
N2—Dy1—N1—C12	−2.2 (4)	O1—S1—C13—C18	87.8 (5)
O6^i^—Dy1—N2—C10	79.8 (5)	O3—S1—C13—C14	149.5 (5)
O2w—Dy1—N2—C10	−80.5 (5)	O2—S1—C13—C14	28.5 (5)
O1w—Dy1—N2—C10	128.8 (5)	O1—S1—C13—C14	−90.7 (5)
O1—Dy1—N2—C10	−131.8 (5)	C18—C13—C14—C15	4.6 (9)
O4^ii^—Dy1—N2—C10	−7.5 (6)	S1—C13—C14—C15	−176.8 (4)
O5^ii^—Dy1—N2—C10	−1.5 (5)	C13—C14—C15—C16	−1.0 (9)
N1—Dy1—N2—C10	177.9 (6)	C13—C14—C15—C19	178.0 (5)
O6^i^—Dy1—N2—C11	−95.5 (4)	C14—C15—C16—C17	−4.3 (8)
O2w—Dy1—N2—C11	104.2 (4)	C19—C15—C16—C17	176.6 (5)
O1w—Dy1—N2—C11	−46.6 (5)	C14—C15—C16—C20	175.7 (5)
O1—Dy1—N2—C11	52.9 (5)	C19—C15—C16—C20	−3.3 (8)
O4^ii^—Dy1—N2—C11	177.1 (4)	C15—C16—C17—C18	6.2 (8)
O5^ii^—Dy1—N2—C11	−176.8 (4)	C20—C16—C17—C18	−173.8 (5)
N1—Dy1—N2—C11	2.6 (4)	C14—C13—C18—C17	−2.7 (9)
C12—N1—C1—C2	−0.6 (10)	S1—C13—C18—C17	178.7 (4)
Dy1—N1—C1—C2	177.2 (6)	C16—C17—C18—C13	−2.8 (9)
N1—C1—C2—C3	0.1 (12)	Dy1^ii^—O4—C19—O5	−3.1 (6)
C1—C2—C3—C4	0.5 (12)	Dy1^ii^—O4—C19—C15	177.0 (5)
C2—C3—C4—C12	−0.5 (10)	Dy1^ii^—O5—C19—O4	3.1 (6)
C2—C3—C4—C5	179.7 (7)	Dy1^ii^—O5—C19—C15	−177.0 (5)
C3—C4—C5—C6	178.4 (7)	C14—C15—C19—O4	16.2 (9)
C12—C4—C5—C6	−1.4 (10)	C16—C15—C19—O4	−164.7 (6)
C4—C5—C6—C7	0.2 (11)	C14—C15—C19—O5	−163.7 (5)
C5—C6—C7—C8	−178.6 (8)	C16—C15—C19—O5	15.3 (8)
C5—C6—C7—C11	1.6 (10)	Dy1^iii^—O6—C20—O7	166.1 (10)
C11—C7—C8—C9	0.4 (12)	Dy1^iii^—O6—C20—C16	−6.8 (16)
C6—C7—C8—C9	−179.4 (8)	C17—C16—C20—O7	−82.9 (7)
C7—C8—C9—C10	0.3 (13)	C15—C16—C20—O7	97.1 (7)
C11—N2—C10—C9	0.5 (11)	C17—C16—C20—O6	90.4 (6)
Dy1—N2—C10—C9	−174.9 (6)	C15—C16—C20—O6	−89.6 (7)

Symmetry codes: (i) *x*+1/2, −*y*+1/2, *z*+1/2; (ii) −*x*+1, −*y*+1, −*z*; (iii) *x*−1/2, −*y*+1/2, *z*−1/2.

### Hydrogen-bond geometry (Å, º)

**Table:**

*D*—H···*A*	*D*—H	H···*A*	*D*···*A*	*D*—H···*A*
O1*w*—H11···O5^i^	0.84 (1)	1.98 (1)	2.817 (6)	175 (7)
O1*w*—H12···O7^iv^	0.84 (1)	1.94 (2)	2.760 (6)	166 (6)
O2*w*—H21···O2	0.84 (1)	1.96 (3)	2.744 (7)	156 (7)
O2*w*—H22···O3*w*	0.84 (1)	1.83 (2)	2.66 (1)	169 (7)
O3*w*—H31···O7^ii^	0.84 (1)	2.05 (1)	2.81 (1)	151 (2)
O3*w*′—H33···O7^ii^	0.84 (1)	2.05 (1)	2.74 (2)	139 (2)
O4*w*—H41···O2^v^	0.84 (1)	2.11 (5)	2.897 (8)	155 (11)
O4*w*—H41···O2^v^	0.84 (1)	2.11 (5)	2.897 (8)	155 (11)
O4*w*—H42···O3^vi^	0.84 (1)	2.04 (6)	2.787 (9)	148 (11)

Symmetry codes: (i) *x*+1/2, −*y*+1/2, *z*+1/2; (ii) −*x*+1, −*y*+1, −*z*; (iv) −*x*+1, −*y*, −*z*; (v) −*x*+1/2, *y*+1/2, −*z*+1/2; (vi) *x*, *y*+1, *z*.
